# A dataset of paired smartphone and BioPlux accelerometer measurements for vertical jump analysis

**DOI:** 10.1038/s41597-026-07367-0

**Published:** 2026-05-06

**Authors:** Youssef Radid, Nuno M. Garcia, Saif Al-jumaili, Frederico Branco, Paulo Jorge Coelho, Ivan Miguel Pires

**Affiliations:** 1 Instituto de Telecomunicações, University of Beira Interior, Covilhã, Portugal; 2 Institute of Biophysics and Biomedical Engineering (IBEB), Faculty of Sciences, University of Lisbon, Lisbon, Portugal; 3 AquaValor—Centro de Valorização e Transferência de Tecnologia da Água, Chaves, Portugal; 4 Institute for Systems and Computer Engineering, Technology and Science (INESC TEC), 4200-465 Porto, Portugal; 5 School of Science and Technology, Universidade de Trás-os-Montes e Alto Douro, 5000-801 Vila Real, Portugal; 6 School of Technology and Management, Polytechnic University of Leiria, Leiria, Portugal; 7 Institute for Systems Engineering and Computers at Coimbra, Coimbra, Portugal; 8 Instituto de Telecomunicações, Escola Superior de Tecnologia e Gestão de Águeda, Universidade de Aveiro, Águeda, Portugal

## Abstract

This study presents and validates a dataset designed to evaluate the accuracy of a smartphone application for measuring vertical jump time. A total of 550 trials were recorded, with jump flight time simultaneously measured by a smartphone (Android) and a reference wearable accelerometer (BioPlux). Two predictive models, Least Squares (LSQ) and Multilayer Perceptron (MLP), were trained to estimate BioPlux flight time from smartphone readings. The LSQ model achieved a mean error of 0.43% and a mean absolute error of 5.32%, while the MLP model obtained 1.2% and 5.36%, respectively. Both models showed low average percentage error relative to the reference system. This work provides a robust dataset and modeling framework for evaluating low-cost, mobile-based movement assessment tools, with applications in neurology, rehabilitation, and sports biomechanics.

## Background & Summary

Vertical jump performance is increasingly recognized as an indirect marker of neuromuscular function, coordination, and motor control, providing valuable insights into motor impairments and rehabilitation progress^1^. Jump flight time, defined as the time between take-off and landing, is a practical metric that reflects these abilities^2^. Traditional laboratory-based systems, such as force plates, provide accurate flight-time measurements but are costly, require specialized environments, and are not easily scalable for routine clinical use^3^.

The emergence of mobile health technologies offers a promising alternative for performance monitoring. Modern smartphones, equipped with high-quality inertial sensors, can estimate jump flight time with minimal additional hardware^4,5^. Previous studies have investigated the feasibility of mobile-device-based jump assessments^1,6^. However, validation against gold-standard wearable systems remains essential before clinical adoption. Notably, White *et al*.^7^ demonstrated that a single body-worn accelerometer combined with machine learning can accurately infer jump performance metrics, reinforcing the potential of portable sensor-based biomechanical analysis. The present work extends this validation to smartphone-based systems, offering a more accessible and scalable approach to jump performance monitoring.

Publicly available datasets and validation studies related to jumping performance have increased in recent years, but they differ substantially in sensing modality, target outcome, and level of data availability. Prior work has examined smartphone-based estimation of jump performance metrics, including flight time and jump height, mainly in app-validation settings against force platforms or other laboratory references^1,2,6,8,9^. Other studies have used wearable inertial sensors and machine learning to estimate jump-related biomechanical outcomes, such as peak power or jump length, from body-worn sensors^7,10,11^. More recently, smartphone sensors have also been validated for landing and stabilization tasks, showing that mobile inertial measurements can provide clinically relevant timing and movement features outside laboratory environments^12^. However, most existing studies focus on model validation or derived performance metrics rather than releasing synchronized raw data from both a smartphone and a reference wearable system. The present dataset addresses this gap by providing paired raw smartphone and BioPlux recordings for 550 jump trials, together with participant metadata, derived flight-time estimates, and benchmark regression outputs.

This study addresses this need by validating an Android smartphone application against the BioPlux wireless accelerometer, part of the Biosignalsplux platform^13^, widely used in biomechanics and rehabilitation research for its reliability. Both systems recorded jump flight time simultaneously under the same conditions.

The dataset comprises 550 trials in which participants performed multiple vertical jumps. Demographic data include participant height (primarily males at 170–175 cm and females at 152–153 cm). For each trial, paired flight time values were recorded: one measured by an Android application used for the research and the other by the BioPlux system, both expressed in milliseconds.

To estimate BioPlux jump flight time from Android measurements, we applied two models: Least Squares (LSQ) and Multilayer Perceptron (MLP). Both models demonstrated high performance, with negligible systematic bias. Error metrics, including mean error, mean absolute error, standard deviation, maximum error, and minimum error, were computed to evaluate performance. These results align with prior work validating mobile-device-based jump measurements^1,6^.

The Accelerometer Data Collected during Jumping Activity dataset includes raw motion data containing detailed Android and BioPlux recordings, enabling further research and model development. The broader significance of this dataset lies in its potential to expand the role of smartphones as reliable, low-cost tools for clinical movement assessment. In neurology, such tools could assist in diagnosing and monitoring motor disorders, enable non-invasive tracking of rehabilitation, and broaden access to functional mobility evaluation in everyday settings^14,15^. Integrating validated sensor-based measurements into devices already used by patients can advance preventive medicine and personalized care^16^.

In conclusion, this dataset provides a validated benchmark for predicting BioPlux jump flight time from Android-based measurements using LSQ and MLP models, bridging the gap between laboratory-grade wearable sensors and accessible mobile tools. It contributes to advancing research in neurology, rehabilitation, biomechanics, and digital health innovation^1,10,11^.

## Methods

### Dataset Description

Each participant performed multiple vertical jumps, during which a smartphone application running on a single device model—the BQ Aquaris 5.7 with Android 4.2, equipped with a quad-core CPU and 16 GB of internal memory and the BioPlux accelerometer were placed at the waist to record flight time simultaneously. The BioPlux device, considered the gold standard, collected data at a sampling rate of 1 kHz and transmitted it wirelessly via Bluetooth to a nearby computer.

The dataset presented in this paper is available in a Mendeley Data repository^17^. The repository contains 550 trial folders, each corresponding to one synchronized jump acquisition. Every trial folder includes exactly three files: (i) BioPlux.txt, containing the raw BioPlux signals for that trial; (ii) Mobile.txt, containing the raw smartphone accelerometer data for the same trial; and (iii) user_data.json, containing the participant metadata and trial-specific derived information. In addition to these 550 folders, the repository root contains one summary spreadsheet, results.xlsx, which consolidates all valid trials, model predictions, error metrics, and trial labels.

Before the experiments, all participants provided written informed consent so we could disclose the test results anonymously. The agreement also required participants to provide informed consent regarding the risks and objectives of the study. Only data about participants who signed a consent to participate in the study were recorded. Participants were also notified that their data would be included anonymously in Mendeley Data. The study was approved by the Ethics Committee of the Universidade da Beira Interior with the reference CE-UBI-Pj-2020-035.

### Sensor Model and Precision

The experimental setup combined two measurement systems: the BioPlux device and the internal sensors of an Android smartphone application developed for jump analysis. The BioPlux platform served as the gold standard reference, while the smartphone application provided a practical mobile-based estimation.

BioPlux integrates the following sensing components:

- **Triaxial Accelerometer**: mounted at the participant’s waist to capture vertical acceleration during take-off and landing. Sampling frequency: 1 kHz, providing high temporal resolution.
- **Pressure Sensor**: embedded in a jump platform to detect foot contact and airborne phases with high precision. This sensor defines the start and end of flight and serves as the reference for validating accelerometer-based estimations.
- **Bluetooth Transmission Module**: ensures real-time data transfer to a nearby computer for storage and processing.

Regarding the Android device, we considered only the use of the **Built-in Triaxial Accelerometer (LIS3DHTR model)** placed at the waist, recording raw acceleration signals during jumps. The sensor sampled data at 100 Hz along the X, Y, and Z axes, with a measurement range of 0–32 m/s^2^, a resolution of 0.004 m/s^2^, and a power consumption of 0.13 mA. No dedicated calibration procedure was applied beyond the manufacturer’s factory settings, and a 10 second stabilization delay was introduced before each recording window to ensure signal reliability^18^.

### Mobile application and algorithm

#### Mobile Application

The application was developed for Android devices (Android 2.3 Gingerbread and above) to measure vertical jump flight time using embedded sensors. The design emphasized a user-friendly interface with minimal interaction. After pressing the start button, a 5-second delay allowed the user to position the device, followed by a beep signaling the jump. Data collection stopped either when the user pressed the stop button or automatically after 10 seconds. All jumps were stored locally, and the application displayed both the most recent and historical results, enabling users to track performance over time. The method implemented in the application follows the approach described in a previous study^1^.

#### Android App Adaptation to Python

To enable large-scale processing of the dataset, the original Android jump-flight-time detection algorithm was reimplemented in Python (version 3.10.4). While the Android application was designed for real-time, user-friendly interaction, the Python version was optimized for automated batch analysis, enabling the processing of hundreds of jump trials in a reproducible, efficient manner. Additionally, the Python implementation facilitates data cleaning, integration with participant metadata, and the generation of visualizations.

The jump detection algorithm relies on peak detection applied to the norm of the tri-axial acceleration vector rather than to a single axis. Vertical jumping generates distinct acceleration peaks associated with take-off and landing, which act as biomechanical landmarks for segmenting the movement and estimating flight time. A peak-detection approach was therefore adopted because it is computationally efficient and well suited for short, ballistic movements such as vertical jumps, making it appropriate for real-time mobile processing.

The acceleration norm, computed as $a(t)=\sqrt{{a}_{x}{(t)}^{2}+{a}_{y}{(t)}^{2}+{a}_{z}{(t)}^{2}}$, was used instead of only the vertical component because the smartphone, positioned in the waist, does not maintain a fixed orientation relative to the body during movement. Rotations of the device and inter-participant variability in placement make axis alignment unreliable. Using only the vertical axis would therefore lead to inconsistent peak amplitudes and reduced detection robustness. The norm-based representation provides orientation-independent features and improves the stability of take-off and landing detection across trials and users.

The algorithm follows the same logic as the original mobile application:

1. **Peak Detection:** Identify the three most prominent peaks in the acceleration magnitude.
2. **Minima Detection:** Between these peaks, locate two local minima corresponding to take-off and landing.
3. **Flight Time Calculation:** Compute the interval between these minima as the jump flight time.

**Fig. 1:**
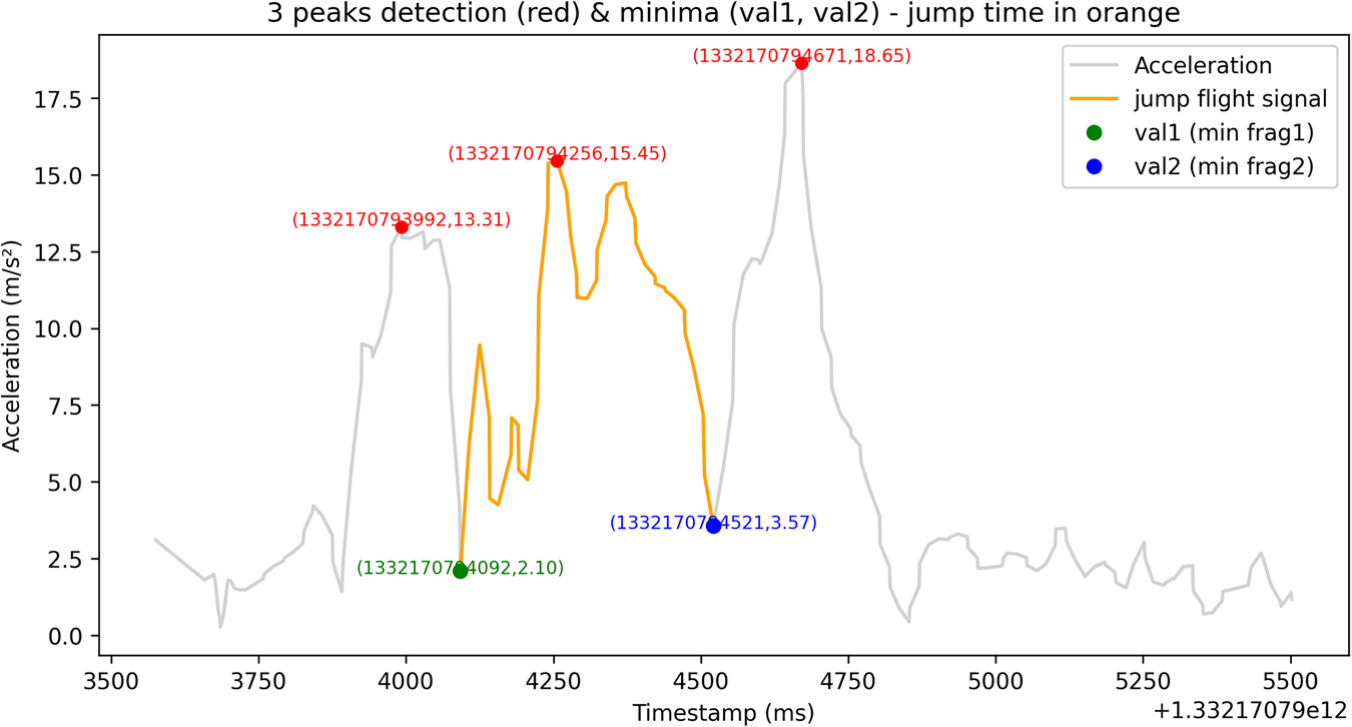
Accelerometer norm signal for the first trial, computed from the smartphone triaxial acceleration data as $a(t)=\sqrt{{a}_{x}{(t)}^{2}+{a}_{y}{(t)}^{2}+{a}_{z}{(t)}^{2}}$. The three main peaks are highlighted in red, the two local minima in green and blue, and the estimated jump flight time in orange. The numeric labels next to each point indicate the timestamp (x-coordinate) and acceleration norm value (y-coordinate, in m/s^2^).

The Python implementation enables direct visualization of the jump detection process based on the norm of the triaxial accelerometer signal, defined as $a(t)=\sqrt{{a}_{x}{(t)}^{2}+{a}_{y}{(t)}^{2}+{a}_{z}{(t)}^{2}}$. This visualization is essential for validating the algorithm’s accuracy and identifying irregular or invalid jumps. Figure 1 illustrates the accelerometer norm for the first trial, where the three most prominent peaks are highlighted in red and the two local minima in green and blue. The time difference between these two minima defines the jump flight time:

$${T}_{{\rm{flight}}}={t}_{{\rm{val2}}}-{t}_{{\rm{val1}}}$$

The orange segment represents the corresponding jump flight interval. The numeric labels next to each marker indicate the timestamp (x-axis, in ms) and the accelerometer norm value (y-axis, in m/s^2^) at each identified peak or local minimum.

### Models for Prediction

Two models were implemented to predict the BioPlux-measured jump flight time using the smartphone-measured jump flight time and user attributes (age, weight, height, and gender).

#### Least Squares (LSQ)

A classical linear regression model was used to estimate a direct linear relationship between the smartphone flight time and the BioPlux reference. The LSQ model assumes a linear relationship between the input and the target and therefore provides a baseline for performance comparison.

#### Multilayer Perceptron (MLP)

To capture non-linear dependencies, a neural network model was trained using the same input variables. The chosen architecture consisted of three hidden layers with 64, 32, and 16 neurons, respectively, with ReLU activation functions, and a regularization parameter alpha = 0.01. Training was performed using a maximum of 1000 iterations and a fixed random state to ensure reproducibility.

The dataset was randomly split into 80% for training and 20% for testing. The models were evaluated using the Mean Squared Error (MSE) and the Coefficient of Determination (*R*^2^).

The model inputs included participants’ age, weight, height, Android jump flight time, and gender, which were chosen because they are known to influence jump performance. All continuous input variables were standardized to have zero mean and unit variance, ensuring that features with different scales contributed equally to model training. Gender was encoded as a binary variable (1 = male, 0 = female). Standardizing the inputs also improves the convergence and stability of learning algorithms. A fixed random seed of 42 was used to guarantee reproducibility of model results across runs.

## Data Records

### Repository structure

The dataset is publicly available on Mendeley Data^17^. At the top level, the repository contains 550 folders corresponding to individual trials, plus one spreadsheet file (results.xlsx) and optional documentation files. Each trial folder contains the raw BioPlux and smartphone recordings and one JSON metadata file.

- BioPlux.txt: text file containing the accelerometer data recorded by the BioPlux Research device for the corresponding jump trial.
- Mobile.txt: text file containing the raw accelerometer data recorded by the Android smartphone application for the same jump trial.
- user_data.json : JSON file that summarizes participant characteristics and links them to the recorded trials. Each file corresponds to a trial and includes at least the following fields: trial number, age, weight, height and gender, as illustrated in Table 1.
- results.xlsx: Excel file that compiles, for each valid trial, the smartphone measured jump flight time, the BioPlux reference value, the predictions of the LSQ and MLP models, the associated error metrics, and a label indicating whether the trial is valid, invalid, or an outlier. Tables 3 and 4 show representative excerpts from this file.
- Optional documentation files (for example, a README or description file) that briefly explain the folder structure, file formats, and how trial numbers map between raw data and the tabular summaries.

This structure allows users to work at multiple levels of analysis. At the signal level, researchers can directly process the raw accelerometer data stored in Bioplux.txt and Mobile.txt for each trial. At the metadata level, the user_data.json files provide participant information and derived trial metrics, such as jump flight time, enabling statistical analyses, comparisons, and model benchmarking without needing to handle the raw sensor signals.

### BioPlux data format

The BioPlux device recorded raw inertial and pressure data for each trial, stored in a tab-separated text file named Bioplux.txt. Each row corresponds to a single time sample, and each column represents a distinct acquisition channel. The file contains eleven columns in total, of which five are relevant to this study.

The first column represents the sample sequence number, serving as a time index for the trial. Columns 4, 5, and 6 correspond to the raw accelerometer readings along the X, Y, and Z axes, respectively. Column 7 contains the pressure signal, which is used to detect ground contact events and derive the jump flight time. The remaining channels were not used in this work.

Example data:

**Table:** 

7	0	0	793	630	734	1001	0	0	0	0
8	0	0	794	631	733	1001	0	0	0	0

Column description:

1 - Sample sequence number (time index)

2 - Unused channel

3 - Unused channel

4 - Accelerometer X-axis (raw)

5 - Accelerometer Y-axis (raw)

6 - Accelerometer Z-axis (raw)

7 - Pressure signal (ground contact detection)

8 - Unused channel

9 - Unused channel

10 - Unused channel

11 - Unused channel

### Android data format

The accelerometer data, which captures Timestamps and acceleration along the X, Y, and Z axes, as well as the overall acceleration magnitude, provides insights into the participant’s movement.

- First column: Timestamp of each sample (ms)
- Second column: acceleration along the x-axis, *a*_*x*_(*t*), in m/s^2^
- Third column: acceleration along the y-axis, *a*_*y*_(*t*), in m/s^2^
- Fourth column: acceleration along the z-axis, *a*_*z*_(*t*), in m/s^2^

The smartphone was attached in the front pocket of the participant’s pants. The orientation was upright in the front pocket, with the device moving naturally with the torso during the jump. This allowed the mobile app to detect the take-off, flight, and landing phases using accelerometer data. The norm of the 3D acceleration vector is computed as:

- Fifth column: norm of the 3D acceleration vector, computed as $a(t)=\sqrt{{a}_{x}{(t)}^{2}+{a}_{y}{(t)}^{2}+{a}_{z}{(t)}^{2}}$, where *a*_*x*_(*t*), *a*_*y*_(*t*), and *a*_*z*_(*t*) are the accelerations measured along the three smartphone axes at time *t*.

Example data:

**Table:** 

Timestamp:	X:	Y:	Z:	Acceleration:
1332246145122	-0.24456802	0.27724624	-0.6322708	0.7324243
1332246145140	-0.33000094	0.20078516	-0.01144886	0.3864536

### User Metadata

Most of the records were obtained from male participants with heights ranging from approximately 170 to 175 cm, while a smaller subset corresponds to female participants with heights around 152 to 153 cm. In addition, the smartphone application stored participant information, such as weight and gender, which complements the raw sensor data. However the study did not specifically analyze the influence of gender on jump flight time or model performance.

The number of trials per participant was not fixed and depended on individual availability during data collection sessions. As a result, it contains an uneven distribution of trials across participants. This distribution was aleatory and does not affect the validity of the recorded signals, as each jump represents an independent measurement. Participant identifiers are provided to allow users to account for subject distribution in subsequent analyses if required.

Across the 550 recorded trials, the associated participant metadata showed a trial-weighted mean age of 22.83 ± 2.59 years, a mean body mass of 76.36 ± 5.38 kg, and a mean height of 169.89 ± 3.46 cm. Most trials were contributed by male participants, with a smaller subset contributed by female participants. Because the number of trials per participant was not uniform, these descriptive statistics should be interpreted as trial-weighted summaries of the recorded dataset rather than as subject-level population estimates. Table 1 provides an overview of participant metadata, highlighting how user information is structured and linked to each recorded jump trial.

**Table 1:** Summary of participant metadata across all trials from JSON files.

Trial Number	Age	Weight (kg)	Height (cm)	Gender
1 .. 50	31	70	175	Male
122 .. 125	23	58	152	Female
126 .. 139	22	48	153	Female
51 .. 121	22	78	170	Male
140 .. 550	22	78	170	Male

### Derived results

In addition to the raw accelerometer recordings, the repository includes a results file that compiles all processed variables and model outputs for each trial. These derived results provide a direct way to analyze the relationship between the smartphone measurements and the BioPlux reference values without requiring users to reimplement the full preprocessing pipeline.

The file results.xlsx contains one row per trial and includes the following fields:

- **Trial Number**: numerical identifier matching the raw data files.
- **Age, Weight, Height, Gender**: participant metadata associated with each trial, taken from the user data file.
- **Android measured value (ms)**: jump flight time estimated by the Android application after applying the detection algorithm.
- **BioPlux measured value (ms)**: reference jump flight time extracted from the BioPlux pressure platform and accelerometer data.
- **Predicted LSQ (ms)**: flight time predicted by the least squares model using smartphone measurements and participant attributes as inputs.
- **Predicted MLP (ms)**: flight time predicted by the multilayer perceptron model using the same input variables.
- **Error metrics**: for each model, the dataset includes the trial-level percentage error and absolute percentage error. These values quantify how closely the predicted flight time matches the BioPlux reference.
- **Comment**: indicates whether the trial is considered valid, invalid, or an outlier based on the criteria detailed in the Technical Validation section.

This file represents the cleaned and consolidated version of the dataset. Only trials labelled as valid are retained, resulting in 516 observations after the removal of invalid trials and statistical outliers, as described in the Technical Validation section.

Because the results file integrates raw measurements, participant metadata, preprocessing labels, and model predictions, it serves as a ready-to-use benchmark for evaluating new regression models, testing alternative outlier-detection methods, or performing statistical analyses of jump flight time estimation accuracy.

## Technical Validation

### Data Cleaning

**Fig. 2:**
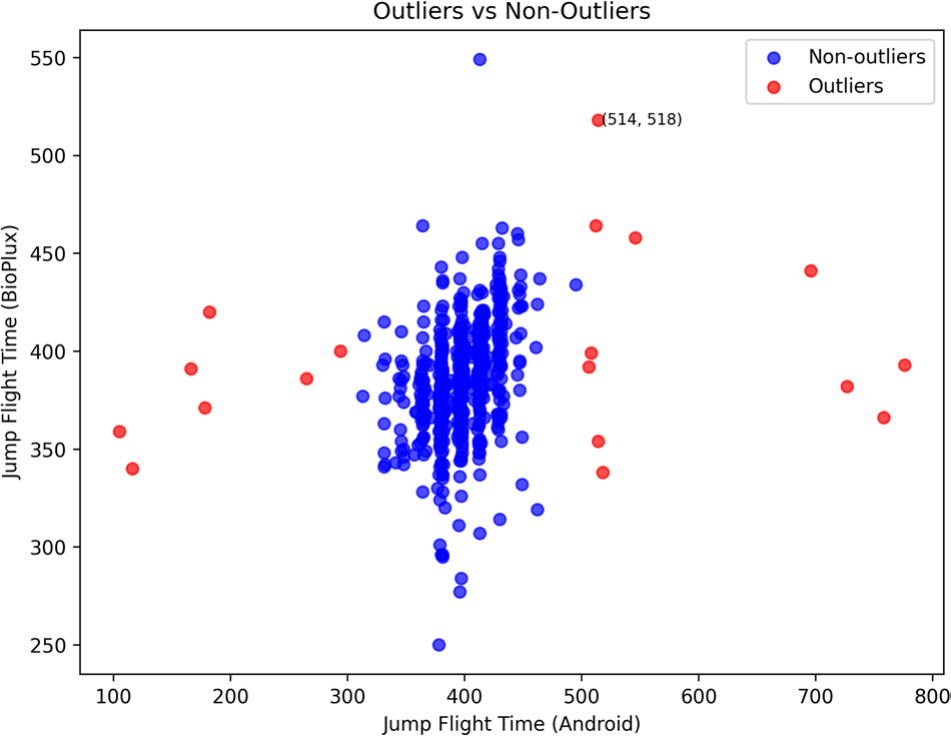
Detection of Outliers and Non-Outliers in Jump Flight Time Measurements.

The initial dataset contained all jump trials recorded by BioPlux and the smartphone application. A total of 16 out of 550 trials were manually removed due to invalid Android jump flight times. These trials showed either physically implausible flight durations (e.g., <150 ms or >700 ms) or extreme discrepancies relative to the reference BioPlux system (>300 ms), indicating sensor malfunction or erroneous detection of take-off/landing events. Subsequently, statistical outlier detection was applied using the Z-score method (|Z| > 2). A total of 18 trials were identified as outliers and removed, leaving a cleaned dataset with 516 valid observations. The distribution of outliers versus non-outliers is illustrated in Fig. 2, which visualizes the detected anomalies based on the Android-estimated jump flight times.

### Outliers and Threshold Justification

As shown in Fig. 2 of the outlier detection analysis, the point (514, 518) was selected as an example. This point (514, 518) was identified as an outlier because its value in the Android column (514) deviates significantly from the overall distribution of jump flight times. The mean of Jump Flight Time (Android) is approximately 399.32 ms, and the standard deviation is 48.64 ms. The corresponding z-score is computed as:

$$z=\frac{x-\mu }{\sigma }=\frac{514-399.32}{48.64}\approx 2.36$$

Since the absolute value of this z-score (|z| = 2.36) exceeds the chosen threshold, the observation is classified as an outlier. A similar reasoning applies to the Bioplux jump flight time value (518 ms, z = 2.44). A threshold of z = 2 was selected to identify points that are more than two standard deviations away from the mean. This criterion provides a balanced trade-off between:

- Detecting anomalous readings caused by measurement noise or sensor inaccuracy.
- Retaining enough valid samples for robust model training.

If a stricter threshold (*z* ≥ 3 or *z* < 2) were used:

- Fewer outliers would be detected,
- But more extreme values (like 514 or 518) would remain in the dataset,
- Leading to a higher Mean Absolute Error (≈ +6%),
- A degradation in model performance, as reflected by higher Mean Squared Error (MSE) and lower R^2^ values.

It occurs because extreme points distort the relationship between the smartphone and reference device readings, making it harder for both predictive models (LSQ and MLP) to generalize accurately.

### Model Performance and Evaluation

Both models were trained on the cleaned dataset, and their performance was evaluated using standard regression metrics, including the Mean Squared Error (MSE), the Coefficient of Determination (*R*^2^), and the root mean-squared error (RMSE). The (*R*^2^) metric quantifies the proportion of variance in the observed data explained by the model, with values closer to 1 indicating a better fit. The mean-squared error (MSE) represents the average of the squared differences between the predicted and actual values, while its square root yields the root mean square error (RMSE), which provides an interpretable measure of prediction error in the same units as the target variable. Because (RMSE) penalizes larger errors more heavily, it is beneficial for detecting performance degradation caused by outliers.

$${R}^{2}=1-\frac{{\sum }_{i=1}^{m}{({y}_{i}-{\widehat{y}}_{i})}^{2}}{{\sum }_{i=1}^{m}{({y}_{i}-\bar{y})}^{2}}\qquad MSE=\frac{1}{m}\mathop{\sum }\limits_{i=1}^{m}{({y}_{i}-{\widehat{y}}_{i})}^{2}\qquad RMSE=\sqrt{MSE}=\sqrt{\frac{1}{m}\mathop{\sum }\limits_{i=1}^{m}{({y}_{i}-{\widehat{y}}_{i})}^{2}}$$

In these equations, *y*_*i*_ represents the true value, ${\widehat{y}}_{i}$ represents the predicted value, and $\bar{y}$ denotes the mean of the observed values.

The Linear (LSQ) model achieved a Mean Squared Error (MSE) of 773.55, a Root Mean Squared Error (RMSE) of 27.81, and a coefficient of determination (R^2^) of 0.16, while the MLP model obtained an MSE of 774.85, an RMSE of 27.83, and an R^2^ of 0.15. These results indicate that both models provide comparable predictive performance, with the LSQ model slightly outperforming the MLP in terms of overall fit. The comparative results of MSE, RMSE, and R^2^ for both models are summarized in Table 2.

**Table 2:** Comparative performance metrics of LSQ and MLP models.

Model	MSE	RMSE	R^2^
Linear (LSQ)	773.55	27.81	0.16
MLP	774.85	27.83	0.15

Tables 3 and 4 present a sample of the data extracted from the results Excel file, this file includes participant characteristics (age, weight, height, gender), measured jump flight times from both the smartphone and BioPlux reference system, the predicted values of each model, the corresponding metric calculations, and a comment indicating whether a given trial is considered valid, invalid or an outlier.

**Table 3:** Extract of 10 trials from the results file showing LSQ model predictions and related error metrics.

Trial Number	Age	Weight (kg)	Height (cm)	Gender	Android measured value (ms)	BioPlux measured value (ms)	Pred. LSQ (ms)	Mean Err. LSQ (%)	MAE LSQ (%)	Comment
1	31	70	175	Male	429	455	396.10	−0.13	0.13	Valid
**28**	**31**	**70**	**175**	**Male**	**1174**	**365**	—	—	—	**Invalid**
29	31	70	175	Male	398	400	382.92	-0.04	0.04	Valid
30	31	70	175	Male	416	393	390.58	-0.01	0.01	Valid
*31*	*31*	*70*	*175*	*Male*	*294*	*400*	—	—	—	*Outlier*
369	22	78	170	Male	371	366	376.05	0.03	0.03	Valid
481	22	78	170	Male	358	369	376.93	0.02	0.02	Valid
*523*	*22*	*78*	*170*	*Male*	*105*	*359*	—	—	—	*Outlier*
533	22	78	170	Male	379	370	377.35	0.02	0.02	Valid

**Table 4:** Extract of 10 trials from the results file showing MLP model predictions and related error metrics.

Trial Number	Age	Weight (kg)	Height (cm)	Gender	Android measured value (ms)	BioPlux measured value (ms)	Pred. MLP (ms)	Mean Err. MLP (%)	MAE MLP (%)	Comment
1	31	70	175	Male	429	455	399.77	-0.12	0.12	Valid
**28**	**31**	**70**	**175**	**Male**	**1174**	**365**	—	—	—	**Invalid**
29	31	70	175	Male	398	400	383.18	-0.04	0.04	Valid
30	31	70	175	Male	416	393	390.85	-0.01	0.01	Valid
*31*	*31*	*70*	*175*	*Male*	*294*	*400*	—	—	—	*Outlier*
369	22	78	170	Male	371	366	373.18	0.03	0.03	Valid
481	22	78	170	Male	358	369	367.65	0.02	0.02	Valid
*523*	*22*	*78*	*170*	*Male*	*105*	*359*	—	—	—	*Outlier*
533	22	78	170	Male	379	370	376.57	0.02	0.02	Valid

In addition to MSE, *R*^2^, and RMSE, both models were evaluated using percentage-based error metrics. The LSQ model showed a Mean Error of 0.43% and a Mean Absolute Error of 5.32%, while the MLP model had a Mean Error of 1.2% and a Mean Absolute Error of 5.36%. Maximum and Minimum Errors were similar for both models, indicating comparable overall performance.

Table 5 summarizes these metrics, providing a clear numerical comparison between LSQ and MLP models. This analysis highlights that both models perform similarly, with average absolute percentage errors around 5%, confirming that the smartphone-based estimation remains closely aligned with the reference Bioplux measurements.

**Table 5:** Error Metrics for LSQ and MLP Models.

Linear (LSQ)	
Mean Error:	0.43%
Mean Absolute Error:	5.32%
Standard Deviation of Absolute Error:	5.43%
Maximum Error:	50.50%
Minimum Error:	0.02%

MLP	
Mean Error:	1.23%
Mean Absolute Error:	5.36%
Standard Deviation of Absolute Error:	5.63%
Maximum Error:	50.80%
Minimum Error:	0.01%

Typical jump flight times are around 450–500 ms. To verify the mathematical consistency of these results, we can compute the relative RMSE as a percentage:

$$\,{\rm{Relative\; RMSE}}( \% )=\frac{{\rm{RMSE}}}{{\rm{mean\; true\; value}}}\times 100$$

Using the observed RMSE  ≈ 27.8 ms and assuming an average flight time  ≈ 500 ms:

$$\,{\rm{Relative\; RMSE}}\,( \% )=\frac{27.8}{500}\times 100=5.56 \% $$

This value aligns perfectly with the Mean Absolute Error of approximately 5.3–5.4% reported in the experimental results, confirming complete coherence across both evaluation approaches.

### Interpretation

The comparison between the LSQ and MLP models highlights the impact of model complexity on prediction performance. While the Linear (LSQ) model provides interpretable and stable results, the MLP model, despite its slightly higher MSE and lower R^2^, demonstrates comparable accuracy and maintains low percentage-based errors, confirming the overall reliability of both approaches. The RMSE of approximately 27.8 ms corresponds to an average deviation of around 6% relative to typical jump flight times (≈450–500 ms), indicating satisfactory predictive accuracy for field applications. The consistency between the absolute metrics (MSE, RMSE, *R*^2^) and the relative percentage-based errors (MAE  ≈ 5.3%) supports the robustness of the evaluation, consistent with the view that *R*^2^ provides a reliable measure for assessing regression model performance^19^.

Although the *R*^2^ values remain modest (≈0.15–0.16), suggesting that only a small portion of the variance in jump performance is explained by the models, the low mean and absolute percentage errors demonstrate that the predictions closely align with the reference Bioplux measurements. It indicates that individual variability and sensor noise likely account for most of the unexplained variance^20^. Moreover, the minimal difference between LSQ and MLP performance suggests that non-linear modeling does not substantially improve prediction accuracy in this context, implying that the relationship between smartphone and reference sensor measurements is mainly linear.

The differences between smartphone and BioPlux measurements were generally small, with mean errors of 0.43% (LSQ) and 1.23% (MLP), and mean absolute errors of approximately 5.3–5.4%, confirming that the smartphone estimates closely align with the reference system without systematic bias.

These findings align with previous studies, such as Pires *et al*.^1^ and Balsalobre-Fernández *et al*.^8^, which also reported strong correlations between smartphone-based estimations and laboratory reference systems^9^. Through automated preprocessing (outlier removal, scaling) and dual-model validation, this work establishes a reproducible framework for evaluating sensor-based jump metrics.

Overall, smartphone-based flight time estimation represents a low-cost, portable, and scalable alternative to specialized laboratory systems. However, it remains influenced by factors such as device placement, user variability, and signal noise.

## Usage Notes

The dataset can be used at two complementary levels of abstraction.

At the signal level, users can process the raw accelerometer recordings stored in the BioPlux.txt and Mobile.txt files to develop new jump detection algorithms, compare different filtering or segmentation strategies, or explore alternative definitions of flight time.

At the tabular level, the user_data.json and results.xlsx files provide a convenient starting point for statistical modelling and benchmarking. The former summarizes participant characteristics and the distribution of trials across users. At the same time, the latter contains the cleaned set of valid trials with measured flight times, model predictions, and error metrics.

Users interested in reproducing or extending the analysis can rely on the Jupyter Notebooks provided in the associated GitHub repository, which implement the Android algorithm in Python, perform data cleaning, and train the LSQ and MLP models. For applications where robustness is critical, we recommend working with the cleaned subset of 516 valid trials, as described in the Technical Validation section. Researchers wishing to test alternative cleaning or outlier-detection strategies can start with the complete set of 550 trials and adapt the provided notebooks accordingly.

Because the dataset focuses on a relatively narrow range of ages, heights, and body masses, care should be taken when extrapolating results to populations with substantially different anthropometric characteristics or to alternative sensor placements.

## Data Availability

The data used in this study is available on Mendeley Data at the following repository: https://data.mendeley.com/datasets/cvfy8gtfn9/2.

## Code availability

The repository contains the Jupyter Notebook Python code for adapting the Android application. A second Jupyter Notebook is included to demonstrate the dataset’s cleaning, preprocessing, and model comparison. All files are available in the same repository: https://github.com/youssefradid/TempoVooPy.git.
